# Atlanta Academy of Medicine

**Published:** 1879-11

**Authors:** T. D. Park

**Affiliations:** Reporter


### Reports of Societies.

### ATLANTA ACADEMY OF MEDICINE.

T. D. PARK, M.D., REPORTER.

ATLANTA, GA., Oct. 13, 1879.

The Academy was called to order with the President, J. S. Todd, M.D., in the chair. After dispatching the business of the evening, reports of cases were called for.

Dr. Calhoun reported the case of a gentleman from Florida, aged 35, who came to see him some weeks ago. He had suffered from enlarged tonsils for seventeen years, which had still more enlarged and given him marked trouble in the last two years. The symptoms most troublesome were the nervous, which resembled very much some of those caused by elongated prepuce in children, viz: restlessness, sleeplessness and great difficulty in co-ordinating his movements. All these were increased by any acute inflammation of the tonsils which might and did arise from time to time. He determined to operate on last Saturday week (4th) and on that day the patient felt better than usual. The operation was completed without much hemorrhage, but occasioned great fear in the patient, who was afraid of hemorrhage. Both tonsils were excised and at least two-thirds of each taken out. He kept indoors for two days, and on Tuesday, the third day, the right tonsil had healed over. On Wednesday he spat a little blood, which caused him to apply to the doctor immediately. Examination discovered a small unhealed spot on left tonsil. All passed on well until the next day about 1 o'clock, when hemorrhage returned, and most profusely. The blood spurted from the wound, and so fast did it flow that the man could scarcely empty his mouth by spitting. Tinc. fer. chlor. was applied locally, and would check the flow for a few minutes, but it would recur This was applied at intervals for an hour or two ice being held in the mouth all the while. Then sent for Monsel's solution, but the druggist not having it sent solution of per. oxide iron. This acted comparatively well, but the hemorrhage would still recur every 20 or 30 minutes. Dr. C. left at 8 o'clock, but was called again at 9, the patient, in the meantime, having had two or three of the hemorrhages, which then ceased of its own accord by the time he arrived. He remained with him until 1 o'clock that night, leaving him in charge of Dr. Salm. He had been kept on his back, and when tired of this position turned on to the side, with mouth open, so that air could come in contact with the wound. From these repeated hemorrhages, the constant oozing, and by his abstenance from food for two days, the man was very much prostrated. Some time before leaving him, Dr. C. administered ¼ grain of morphine, when he went to sleep and the hemorrhage ceased. Does not know if morphine was the cause of the checking. Yesterday (Sunday) a small amount oozed from the wound, but to-day it had quite healed over. Thinks that the man lost at least two quarts of blood.

Dr. C. said he had operated very frequently—in over 150 cases—but had never known of excessive hemorrhage to result previously. Did not fear the least danger from hemorrhage, but has reason now to change his opinion. The nervous symptoms had all disappeared before the hemorrhage occurred.

Dr. Scott did not think the hemorrhage was the result of diathesis, as it would have taken place just after or from the time of the operation, while this was delayed for five days. In reply to question by Dr. Baird, Dr. Calhoun mentioned that pulse was very rapid during the evening of the hemorrhage, but that it improved under the morphine.

Dr. Baird was of opinion that some small artery had enlarged with the hypertrophy of the tissue of the tonsil in order to supply it with sufficient blood, and that when it was severed in the operation the pressure of the adjacent tissue and the retraction of its inner coats were sufficient to plug its orifice. That sloughing ensued and secondary hemorrhage was the result. Dr. B. thinks that it might have been checked by the hot iron and ergot given internally. Ergot acts so well in hemorrhages of the lungs, of the uterus, and in congestions that he thinks it would have been indicated in this case.

The recital of this case recalls to the Doctor's mind the details of a case he was called to attend over a year ago. A negro girl had broken a piece of crockery, which had made a deep incised wound in the hand. She had immediately proceeded to a drug store where a very powerful caustic was freely applied. This checked the hemorrhage, but when the time arrived for the slough to separate, secondary hemorrhage occurred, and his call was the consequence. It was late in the evening when he arrived, to find the girl bleeding profusely from the deep, ugly wound in her hand, and also to find that her friends had assisted the flow, as far as lay in their power, by tying a bandage around her forearm. He immediately removed this, and pressing upon the artery above the wrist, endeavored with his forceps to catch hold of the end of the vessel and twist it. But a bad light, a deep, ragged wound, a bevy of female eyes, were not at all conducive to success, and over him would flash the wish that he were some other person just for that short space of time. When he would take the pressure off the artery, out the blood would spurt. and finally in a fit of desperation he took a director from his pocket-case and inserting it into the wound pressed the parts against the bone, moving the director in various ways. The blood ceasing to flow caused, as is supposed, by the director striking the mouth of the vessel and so bruising or twisting it that the flow was checked. Next morning he found her doing well, and from that time had no further trouble.

Dr. Salm reports the following case to obtain the opinion of gentlemen of the Academy:

Some time ago a negro woman consulted him in regard to a tumor on the lobe of each ear. The growths had enlarged to the size of hen eggs, and the history was that two years ago an old negro man had taken off two similar but somewhat smaller ones with his razor. It appears that he was desirous of extirpating the growth permanently, as he took away the whole lobe, using the instrument mentioned for the purpose.

The tumors were successfully removed by Dr. S., and the woman has done well since. He would like to have the opinion of the gentlemen present as to the recurrence or non-recurrence of this class of tumors.

Dr. Calhoun thought that they would be likely to recur in this case, and that recurrence was the rule.

The others acquiesced in the opinion.

Dr. Scott reported case of an old gentleman 55 or 60 years of age, who several months ago contracted what he considered an ordinary acute pharingitis. He used gargles for three months, and at the end of that time consulted Dr. S. Upon examination the entire pharingeal wall was ulcerated, with loss of tissue, and the left tonsil partly destroyed. His hearing was affected and his breath most offensive. The nasal douche, gargles of permanganate of potassium and steam inhalation were prescribed, with strong solution of nitrate of silver applied by Dr S., and for the first ten days he improved very satisfactorily. At this time the Doctor was absent from the city for three days, and upon his return found a thick, tough, whitish membrane covering the diseased surface, and the right tonsil. He could lift the membrane up at the centre and apparently it was very loosely attached at the edges, but this was deceptive. After using the remedies he had just prescribed for a day or two longer without effect, he decided to remove the membrane, and after cutting through the tough leathery coat, found no difficulty in tearing it away, as the tissue underneath was honeycombed and seemed as if everything except the connective tissue had been destroyed. Hydrochloric acid was applied after the operation, which was attended with no hemorrhage, and patient ordered to return next evening. We heard the next morning that he was doing very well, but owing to the terrific stench the belief among his lady friends that he was putrefying was created, and on their advice not to return, he changed to another physician. Also gave up this one after first visit, and has since relied on his own resources. Under the tonics which he has kept up from the first, his health has improved, but the membrane has appeared again in spots. It showed no tendency to spread from its first limits. Was of opinion that it was malignant.

Academy adjourned.

